# Research on Human Travel Correlation for Urban Transport Planning Based on Multisource Data

**DOI:** 10.3390/s21010195

**Published:** 2020-12-30

**Authors:** Hua Chen, Ming Cai, Chen Xiong

**Affiliations:** 1 School of Intelligent Systems Engineering, Sun Yat-sen University, Guangzhou 510275, China; chenh393@mail2.sysu.edu.cn (H.C.); caiming@mail.sysu.edu.cn (M.C.); 2 Guangdong Provincial Key Laboratory of Intelligent Transportation System, School of Intelligent Systems Engineering, Sun Yat-sen University, Guangzhou 510275, China

**Keywords:** urban transport planning, human mobility, data mining, data analysis

## Abstract

With the rapid development of positioning techniques, a large amount of human travel trajectory data is collected. These datasets have become an effective data resource for obtaining urban traffic patterns. However, many traffic analyses are only based on a single dataset. It is difficult to determine whether a single-dataset-based result can meet the requirement of urban transport planning. In response to this problem, we attempted to obtain traffic patterns and population distributions from the perspective of multisource traffic data using license plate recognition (LPR) data and cellular signaling (CS) data. Based on the two kinds of datasets, identification methods of residents’ travel stay point are proposed. For LPR data, it was identified based on different vehicle speed thresholds at different times. For CS data, a spatiotemporal clustering algorithm based on time allocation was proposed to recognize it. We then used the correlation coefficient r and the significance test *p*-values to analyze the correlations between the CS and LPR data in terms of the population distribution and traffic patterns. We studied two real-world datasets from five working days of human mobility data and found that they were significantly correlated for the stay and move population distributions. Then, the analysis scale was refined to hour level. We also found that they still maintain a significant correlation. Finally, the origin–destination (OD) matrices between traffic analysis zones (TAZs) were obtained. Except for a few TAZs with poor correlations due to the fewer LPR records, the correlations of the other TAZs remained high. It showed that the population distribution and traffic patterns computed by the two datasets were fairly similar. Our research provides a method to improve the analysis of complex travel patterns and behaviors and provides opportunities for travel demand modeling and urban transport planning. The findings can also help decision-makers understand urban human mobility and can serve as a guide for urban management and transport planning.

## 1. Introduction

Obtaining urban traffic patterns and the population distribution of urban residents is the basis for urban transport planning. As a result of rapid urbanization and intelligentization, the population distribution, travel demand, and travel characteristics of cities are quickly changing. However, the traditional survey methods can only reflect the traffic characteristics of a city within a fixed period of time. It is difficult to adapt these methods to the rapid development of cities due to their long cycles and low sample sizes. This brings challenges to urban transport planning and management. To fully understand the urban traffic characteristics, urban management departments and service agencies need to acquire urban travel characteristics data at low cost and high frequency to support the needs of urban management and planning. Meanwhile, with the rapid development of information collection technology, the acquisition of large-sample, multi-dimensional, and fine-grained information is becoming easier and easier.

New traffic data collection methods are generally divided into four categories [1]:

(1) Location-based traffic information acquisition techniques, such as floating cars with global positioning systems (GPSs). It is collected by vehicles equipped with GPSs and communication devices driving on the road.
(2) Radio frequency-based traffic information acquisition techniques, such as radio frequency identification (RFID). RFID generally consists of readers, tags, and back-end systems. Each label has a unique identification mark, which is often pasted or bound to the body for tracking and management.
(3) Video-based traffic information acquisition techniques, such as license plate recognition (LPR) systems. LPR data are collected through the smart bayonet system. The smart bayonet system is composed of front-end equipment, communication transmission network and back-end monitoring and management platform. When a vehicle passes through the system, it will be photographed and recorded.
(4) Sensor-based traffic information acquisition techniques, such as microwave radar, inductive loop detectors, and magnetometers. A set of ground-induction coils are buried under the road, and the driving situation of the vehicle is obtained by detecting the change of the coil inductance.

However, data acquired by location-based traffic information acquisition technology, such as GPS data, can only be collected on vehicles equipped with GPSs and communication devices, such as taxis and online car-hailing. RFID data can only be collected by vehicles equipped with RFID equipment. They are relatively small as a sample size. Sensor-based traffic information acquisition techniques, such as inductive loop detectors, can only collect the passing record of the vehicle, but cannot collect any other characteristics, such as vehicle color, license plate number, etc.

In recent years, automatic license plate recognition technology has been actively developed and promoted, and a huge amount of LPR data has been obtained. An LPR system is a system that uses advanced photoelectric technology, image processing technology, and pattern recognition technology to take images of each passing vehicle and automatically recognize vehicle license plates. The LPR system is usually installed a few meters away from the highway. When a vehicle is detected at a certain distance in front of the device, the system begins to capture images of the vehicle. The image recognition algorithm is used to identify the vehicle license plate number, vehicle type, color, and other vehicle information. The data are then stored in the database and finally uploaded to the traffic administration data center [2].

Scholars have conducted research based on LPR data. The LPR system is essentially a network composed of cameras, which can take pictures of each passing vehicle and automatically convert them into a detailed spatiotemporal record to capture vehicle data in real time with high precision and wide coverage. The information in the LPR record includes the detector ID (representing different camera mounts), the license plate number (vehicle ID), the direction of the vehicle, and the time stamp when the camera captured the vehicle. Therefore, the LPR system collects the spatiotemporal information of each vehicle and reconstructs the travel track of each vehicle by linking a series of spatiotemporal records [3]. As an important input for traffic demand management, the method of obtaining the origin–destination (OD) matrix based on LPR data has become a popular research area. In 2000, Dixon et al. proposed a model to estimate the OD information of vehicles based on LPR data. The trajectory data left by the vehicle in the LPR system were used to analyze the travel characteristics, and the OD matrix was obtained, which was verified on a highway [4]. Antoniou et al. directly estimated the dynamic OD using the OD matrix obtained from LPR data [5]. Sun et al. obtained the vehicle’s path node and travel time by recognizing the vehicle license plate, completed the vehicle information with the missing path through the Bayesian estimation model, obtained the initial OD matrix based on the LPR data, and then corrected the initial OD matrix using the road traffic flow to obtain the final OD matrix [6]. Zhou et al. used the vehicle trajectory data obtained by the Chongqing RFID system to obtain vehicle travel OD information and compared the data with resident travel survey data. The vehicle travel stay point recognition, OD segmentation, and the vehicle behavior portrait were used to obtain the vehicle OD [7]. Apart from obtaining the OD, LPR also plays an important role in estimating the traffic flow [8], which is essential for a wide range of intelligent transportation system (ITS) applications, such as carpooling [9], travel behavior clustering [10], queue length estimation [11,12], traffic state estimation [13], and trajectory reconstruction [14]. Thus, it can be seen that LPR has become an indispensable data resource in transportation research and plays a crucial role in promoting the development of intelligent transportation.

Another data source that provides an emerging and promising source of information for urban transport planning is cellular signaling (CS) data. CS data are communication data between mobile phone users and the transmitting base station or microstation. As soon as the mobile phone is turned on, CS data begin to be generated. Due to its large sample size, long observation period, short sampling period, and strong followability, CS data have attracted widespread attention from researchers. The information in a CS data record includes the data record number, base station location area code, traveler ID (unique identity), base station identification code, communication time, GPS longitude, GPS latitude, traveler gender, and traveler age. Therefore, the CS data contain the location information of each user, allowing the trajectories of each traveler to be reconstructed by summarizing a series of spatiotemporal records. Apart from individual mobility research, CS data play an important role in calculating the regional population, estimating OD flows, constructing traveler profiles, and analyzing the spatiotemporal distribution of an urban population.

CS data have stimulated researchers to review the conventional research questions about human mobility at an unprecedented spatiotemporal scale, with contents including, but not limited to, traffic demand analysis and control, dynamic population spatial distribution analysis, road spatiotemporal uniformity management [15,16,17], occupation and residence commuter channel analysis, external passenger flow channel analysis, transport mode detection [18], large passenger flow analysis [19], and urban arterial traffic status detection [20]. An in-depth understanding of human spatiotemporal flow patterns and their interactions with the urban environment can be beneficial for various applications, from urban planning and transportation to public health [21]. The human mobility patterns are closely related to the population distribution and urban traffic patterns. Some attempts have been made to analyze urban traffic. Jiang et al. used CS data to obtain the daily activities and travel characteristics of Singapore residents [22]. Alexander et al. obtained the travel matrix of residents in Boston based on CS data and inferred the travel purpose of residents based on historical information [23]. Gao et al. used CS data to extract the characteristics of the travel time and the space distribution of Beijing residents [24]. Liu et al. analyzed how urban land use influences commuting flows in Wuhan from the perspective of CS data [25].

Based on the above studies, LPR and CS data have become indispensable forms of data in transportation research. However, their research mainly has the following shortcomings: (1) Owing to the costs or difficulty in data acquisition, many scholars’ traffic planning, OD analysis, and other traffic studies were only based on individual datasets. An individual dataset has certain limitations in analyzing traffic patterns and population distributions. (2) Although many scholars have conducted a lot of research using CS and LPR data, they did not distinguish between stay population and move population. In transportation research, the move population and stay population often have different spatial and temporal patterns. Therefore, the identification of the move population and stay population is of great importance in transportation research. (3) Owing to differences in the data collection rules, data density, and data acquisition methods of different data sources, it is difficult to determine whether there are significant differences in urban traffic analysis results obtained using different data sources.

To deal with these problems, the CS and LPR datasets from five working days in Foshan, China, were used to analyze the population distribution and traffic patterns that are of most concern for urban transportation planning. First, based on the different characteristics of CS and LPR data, algorithms were designed to identify the move population and stay population. Then, the move population and stay population distribution were obtained. Finally, the correlation degrees of the results were analyzed in terms of the correlation coefficient and the significance level.

The remainder of this article is organized as follows. The LPR and CS datasets are described in Section 2, respectively. The data preprocessing is also introduced in this section. The LPR- and CS-based stay-point recognition methods are proposed in Section 3, respectively. The correlation indices are also introduced in this section. The results and discussion are presented in Section 4. Finally, Section 5 presents conclusions and future research directions.

## 2. Data Description and Preprocessing

**Figure 1:**
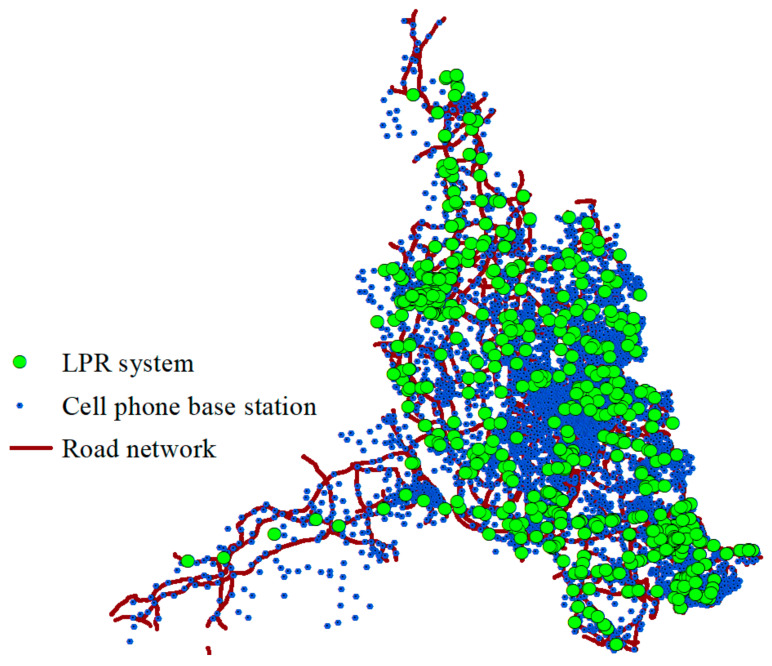
Road network and distribution of license plate recognition (LPR) system and cell phone base station in Foshan.

The study area of this research was Foshan, which is located in the southeastern part of China. Currently, the total area of Foshan is approximately 3800 square kilometers and is divided into five administrative districts, which can be further classified into 32 administrative streets/towns. The permanent population of Foshan is 7.35 million, of which the registered population is 3.85 million. We used the CS and LPR data of five working days from July 16 to 20, 2018, in Foshan to conduct this study. The number of mobile phone base stations of operators providing CS data was about 5657, and the number of LPR systems in Foshan was 1037. The road network and distribution of the LPR systems and base stations in Foshan are shown in Figure 1, in which the lines represent the road network in Foshan, the colored dots represent the LPR systems, and the colored pentagons are cell phone base stations. As shown in Figure 1, the distribution of cell phone base stations and LPR systems covered almost all of Foshan city. Therefore, it is feasible to use the two datasets to analyze urban traffic patterns and the population distribution.

### 2.1. License Plate Recognition (LPR) Data

We used encrypted LPR data collected from Foshan, China. The LPR data are encrypted using MD5 algorithm. It is a hash algorithm. It has two important characteristics: (1) the value of plaintext data after hashing is fixed; (2) the result of hashing any piece of plaintext data must always be the same. In the dataset, each encrypted data point represented a unique license plate number. In summary, the dataset contained over 3.46 million records from 1.49 million vehicles. According to incomplete statistics, by the end of 2018, the number of vehicles in Foshan was over 2.53 million. Thus, the dataset covered over half of the vehicle ownership in Foshan. Table 1 shows the format of the raw LPR data collected by the LPR system in Foshan. As shown in Table 1, the vehicle ID was the unique encrypted identity code of a vehicle. The record time was the exact time at which a vehicle passed. The address represents the installation position of an LPR system. The drive direction indicates the direction in which a vehicle was driving. The detector ID denotes the identifier of the detector. Longitude and latitude represent the specific location of the LPR system.

Owing to multiple factors such as weather, visibility, light, and technology, some dirty data inevitably appeared in the LPR system during collection. These dirty data needed to be pre-processed to improve the accuracy of traffic analysis. The principles of the data preprocessing were as follows:

(1) Only the vehicle ID, record time, longitude, and latitude were reserved for this study.
(2) Records with blank fields were deleted.
(3) Records with the wrong license plate numbers were removed.

### 2.2. Cellular Signaling (CS) Data

The CS data used in our study were acquired from a major mobile phone operator in China. This dataset covered the footprints of nearly 3.27 million mobile phone users within five workdays, which was close to 85% of the registered population and 44.5% of the permanent population in Foshan. It contained more than 2168 million records. Generally, traditional resident travel surveys can only cover about 1% of the urban population [22]. Therefore, the coverage of our data is much larger than that of traditional traffic surveys.

Table 2 shows the format of the raw CS data collected by the mobile operator. The user ID was the unique encrypted identity code of a mobile phone. The cell ID represented the base station identifier. The record time was the exact time of the CS data record. Longitude and latitude represent the position of the corresponding cell phone tower to which the phone was connecting. Age and gender are the personal information of the registered user. For privacy protection, the user ID and cell ID were encrypted before being used for analysis.

Owing to the influence of various factors, such as the weather, obstacle blocking, and signal strength, some dirty data will appear in the CS data, which must be cleaned. Data cleaning mainly included the following steps:

(1) Only the following data fields were retained: user ID, record time, longitude, and latitude. The other fields were removed during preprocessing.
(2) The dirty data of the same user at different locations at the same time were deleted.
(3) Records with blank fields were removed.
(4) For records that appeared continuously in the same position, only the first and last records were kept.

## 3. Methodology

To study the dynamic distribution of urban residents, mobile phone users and vehicles were divided into move and stay states. The move state means that the user was in the process of a trip, while the stay state indicates that the user was staying in a certain location to engage in work, study, leisure, or entertainment activities. In contrast to the moving state, people are usually in the staying state for most of the day. The move population was mainly used to describe the characteristics of population mobility in the region, while the staying population expressed the common characteristics of crowd gathering. By differentiating the spatial and temporal information, we can further understand the differences in the functional use of the city by the population.

### 3.1. Stay Point Recognition Algorithm Based on Different Speed Thresholds

The identification of the stay points of the vehicle using the LPR data can be divided into two cases: (1) the end point of the previous trip (EPPT) and the start point of the next trip (SPNT) are in different LPR systems; (2) the end point of the previous trip and the start point of the next trip are the same LPR system. The identification of the staying locations in these two cases should be handled separately. The basic idea of stay point recognition is to judge whether the vehicle stays between two track points based on its speed [26].

In the first case, the steps to identify the stay point are as follows:

Step 1: First we use ArcGIS path matching algorithm to match the LPR system to the road. Where ArcGIS provides users with a scalable, comprehensive geographic information system platform. We then obtain the shortest path between LPR systems by requesting the Baidu Map API, which is a set of free application interfaces for developers based on the Baidu Map service, to establish the shortest distance matrix of the LPR systems.

Step 2: Based on the time series of the passing records of a vehicle, the timestamp and location of adjacent records are collected, and the driving speed is calculated based on the shortest distance and time difference:

(1) $$v_{drv}=\frac{d_{sht}}{t_{down}-t_{up}}$$

where $v_{drv}$ represents the driving speed of the vehicle, $d_{sht}$ denotes the shortest distance obtained from step 1, and $t_{down}$ and $t_{up}$ are the timestamps of the downstream and the upstream LPR systems, respectively.

Step 3: Based on the travel times between different LPR systems in the statistical time window T, the lower speed limit $v_{lower}$ is calculated, and the lower speed limit matrix is obtained. As the volatility of the traffic flow within a day leads to fluctuations in the travel time, the abnormal value during peak hours may be normal during peak hours. Therefore, a statistical time window was set, and the travel time within the same time window was considered to be stable. We set T from 00:00 to 06:00 as 60 min and T from 06:00 to 24:00 as 30 min. $v_{lower}$ was the maximum value of the lower 5% of the travel speeds between two LPR systems and 5 km/h:

(2) $$v_{lower}=\max\left\{v_{5\%},5\right\}$$

Step 4: If the driving speed $v_{drv}$ between two LPR systems was smaller than $v_{lower}$, we considered the vehicle to have stopped between the two LPR systems and marked the previous LPR system as the stay point; otherwise, it was considered a moving point.

In the latter case, i.e., when the end point of the previous trip and the start point of the next trip are in the same LPR system, if the time difference (TD) between two consecutive records was greater than 20 min, we defined it as a stay point.

The above algorithm can be summarized by Algorithm 1.
**Algorithm 1** Stay point recognition algorithm based on different speed thresholdsInput: LPR data of a vehicle, denoted as $O_{lpr}$Output: LPR Stay points ($O_{sp}$) and Move points ($O_{mp}$)For each point in $O_{lpr}$ do:  If EPPT == SPNT:    If TD > 20 min: $O_{sp}$ ← SPNT    Else: $O_{mp}$ ← SPNT  Else:      If $v_{drv}$ > $v_{lower}$: $O_{mp}$ ← SPNT      Else: $O_{sp}$ ← SPNTEnd for

### 3.2. Spatiotemporal Clustering Algorithm Based on Time Allocation

Different from traditional k-means and density-based spatial clustering of applications with noise (DBSCAN) clustering algorithms, since different mobile phone users have different spatiotemporal travel patterns, we need to first calculate the stay time of a user at a certain base station. If the stay time exceeded the threshold $t_{min}$, the user was considered to have stayed at the base station. Otherwise, the user may just pass by. We need to design an algorithm to determine the potential location of the user. Hence, we proposed a spatiotemporal clustering algorithm based on time allocation to calculate the user’s actual stay position.

When the stay time of a user at a certain base station exceeded the threshold $t_{min}$, the user was considered to have stayed at the base station, that is, the position of the base station was the stay point of the user. When the user’s location switched back and forth between different base stations in a time less than $t_{min}$, it was very likely that the user was slowly moving or staying near the switched base stations, which was a potential stay space–time mode. In this case, we need to calculate the user’s actual stay position, and the calculation formula is as follows:

(3) $$\left\{\begin{array}{c} lng_{new}=\overset{n}{\underset{i=1}{\sum }}\frac{t_{i}}{t_{n}-t_{1}}lng_{i}\\ lat_{new}=\overset{n}{\underset{i=1}{\sum }}\frac{t_{i}}{t_{n}-t_{1}}lat_{i} \end{array}\right.$$

In the formula, $lng_{new}$ and $lat_{new}$ represent the longitude and latitude of the stay point, $t_{1}$ and $t_{n}$ represent the timestamps of the first and last points, respectively, and $t_{i}$ denotes the stay time at the *ith* base station.

Based on Equation (3), we proposed a spatiotemporal clustering algorithm based on time allocation, described as follows:

Step1: The pre-processed CS data were input, with each user as the processing unit. All the records of user *i* in one day were selected and sorted. The base stations passed by the user *i* was named O_1_, O_2_, …, O*n* according to the time sequence.

Step2: Points O_1_ and O_2_ were selected in sequence, and the user’s potential stay point was calculated using Equation (3). We then obtained the new point with longitude $lng_{new}$ and latitude $lat_{new}$. If both the distances from O_1_ to potential stay point O and from O_2_ to O were less than distance threshold $d_{min}$, O_1_ and O_2_ may have constituted a stay. Three consecutive points O_1_, O_2_, and O_3_ were then selected, and the new potential stay point O was recalculated. We then reobtained the new point with longitude $lng_{new}$ and latitude $lat_{new}$. If the distance from O_1_ to O, from O_2_ to O, and from O_3_ to O were less than $d_{min}$, then O_1_, O_2_, and O_3_ may have constituted a stay. By analogy, when *n* consecutive points O_1_, O_2_, …, O*n* were selected, and there was a point whose distance from O was greater than $d_{min}$, the loop stopped.

Step 3: The time interval from O_1_ to O_n−1_ was calculated. If the time interval was greater than the time threshold $t_{min}$, it was then considered to constitute a stay. The longitudes and latitudes of the points O_1_, O_2_, …, O_n−1_ were replaced with the longitude and latitude of O, and they were marked as a stay position.

Step 4: If the time interval was smaller than the time threshold $t_{min}$, it could not constitute a stay. Point O_1_ was marked as a moving point. Two points O_2_ and O_3_ were selected in turn, and the process returned to step 2. The loop was continued until all points of user *i* were traversed.

**Figure 2:**
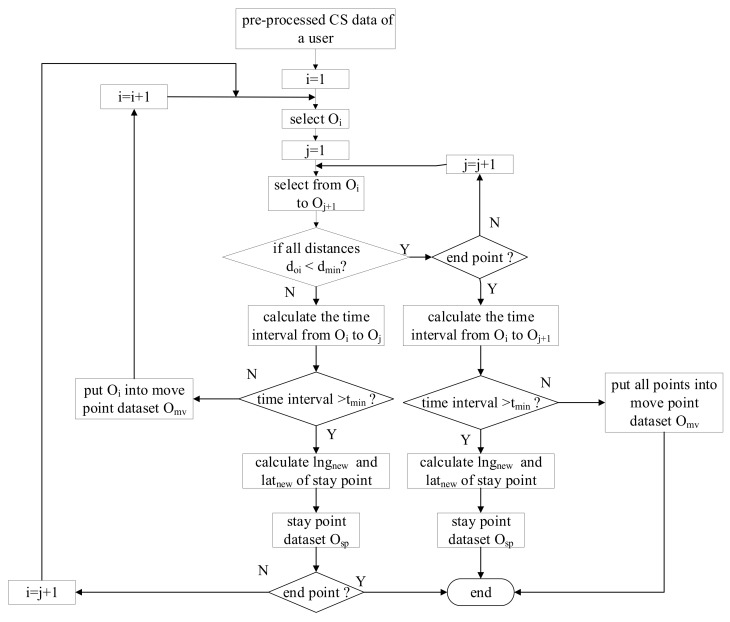
The flowchart of spatiotemporal clustering algorithm based on time allocation.

The above algorithm can be summarized by the following flowchart (see Figure 2).

In Figure 2, O_i_ and O_j+1_ denote the *ith* and *j* + *1th* point of user, respectively. d_oi_ represents the distance from O_i_ to potential stay point O. O_mv_ is the move point dataset and O_sp_ is the stay point dataset. The variables t*_min_* and d*_min_* are the time threshold and distance threshold, respectively. *lng_new_* and *lat_new_* are the longitude and latitude of potential stay point calculated by Equation (3).

### 3.3. Correlation Index

In traffic analysis, the commonly used spatial analysis unit is the traffic analysis zone (TAZ). Thus, it is necessary to count the number of users/vehicles of two types (move and stay) in the TAZ throughout a day. To compare the calculation results of different datasets, the proportion of TAZ users/vehicles was used instead of the absolute number. The calculation method was as follows:

(4) $$P_{i,Type}=\frac{N_{i,Type}}{\sum_{i}^{n}N_{i,Type}},\;Type=\left\{stay,\;move\right\}$$

where $P_{i,Type}$ represents the proportion of users of each type in the TAZ, *Type* is either stay or move, $N_{i,Type}$ represents the number of users of each type in TAZ *i*, and *n* is the number of TAZs.

#### 3.3.1. Correlation Coefficient

To quantitatively describe the differences in the population distribution and urban traffic pattern calculated using the two datasets, the correlation coefficients and significance levels were used as indicators. Correlation is a non-deterministic relation, and the correlation coefficient is one of the indicators used to measure linear correlations between the variables. The Pearson correlation coefficient (defined as r) is used to measure the degree of correlation between two variables. It is generally believed that when 0 < |r| ≤ 0.3, the two variables are weakly correlated; when 0.3 < | r| ≤ 0.5, the two variables are slightly correlated; when 0.5 < |r| ≤ 0.8, the two variables are significantly correlated; when 0.8 < |r| ≤ 1, the two variables are highly correlated. r can be calculated by the following formula:

(5) $$r=\frac{\sum_{i=1}^{n}\left(P_{i,Type,A}-\bar{P_{Type,A}}\right)\left(P_{i,Type,B}-\bar{P_{Type,B}}\right)}{\sqrt{\sum_{i=1}^{n}{\left(P_{i,Type,A}-\bar{P_{Type,A}}\right)}^{2}}\sum_{i=1}^{n}{\left(P_{i,Type,B}-\bar{P_{Type,B}}\right)}^{2}}$$

where r is the Pearson correlation coefficient of the two datasets, *n* denotes the number of TAZs, $P_{i,Type,A}$ and $P_{i,Type,B}$ are the proportion of user types in the TAZs in datasets A and B, respectively, $\bar{P_{Type,A}}$ and $\bar{P_{Type,B}}$ denote the average proportion of user types in the TAZs in datasets A and B, respectively.

#### 3.3.2. Significance Test

The correlation coefficient can only show that there is a correlation between the LPR and CS data in these five working days. Since this is only a sample, there may be systematic sampling errors in the correlation coefficients obtained. When the overall correlation coefficient is 0, the calculated correlation coefficient may not be 0 due to sampling error. Therefore, to judge whether the correlation coefficient is meaningful, it must be compared with the overall correlation coefficient. This requires hypothesis testing on r to determine whether it was caused by sampling errors or there was indeed a correlation between the two variables. A significance test is based on a hypothesis related to the parameters of the population (random variables) or the distribution form of the population formed in advance, and the sample information is used to judge whether the hypothesis is reasonable, i.e., to judge whether the true status of the population is significantly different from the original hypothesis. The significance test of the Pearson correlation coefficient is the *t*-test. Therefore, *t*-test was used in this analysis, and the steps were as follows:

Step 1: A hypothesis was established.

$H_{0}$: r=0, LPR and CS data are linearly independent;

$H_{1}$: r≠0, LPR and CS data linearly related.

Step 2: A significance level was set.

The significance level was established by convention. Generally, α = 0.05, or 0.01. This means that the error rate of the significance test must be less than 5% or 1%, respectively (in statistics, events with a probability of less than 5% in the real world are usually called “impossible events”). We defined a significance level of α = 0.05 in this study.

Step 3: *t*-test statistics were calculated.

The *t*-test statistic can be calculated using the following formula:

(6) $$t=\frac{\left|r\right|\sqrt{n-2}}{\sqrt{1-r^{2}}}$$

By calculating *t*, we can obtain the *p*-value. If the observed value of the statistic *t* obtained from the sample is *t*_0_, then the *p*-value can be obtained by the following formula:

(7) $$p-value=P\left\{\left|t\right|\geq \left|t_{0}\right|\right\}=2*P\left\{t\geq \left|t_{0}\right|\right\}$$

The *p*-value reflects the probability of an event occurring. In statistics, the *p*-value is obtained based on the significance test method. Generally, *p* < 0.05 is considered to be statistically different, *p* < 0.01 is considered to be statistically significant, and *p* < 0.001 is considered to be extremely statistically significant. This means that the probabilities that the difference between samples is caused by sampling error are less than 0.05, 0.01, and 0.001, respectively. The *p*-value cannot assign any importance to the data, only the probability of an event occurring. Since the calculation of the *p*-value is not the focus of this article, readers can refer to the relevant literature [27].

## 4. Results and Discussions

### 4.1. Correlation Analysis of All-Day Population Distribution

**Figure 3:**
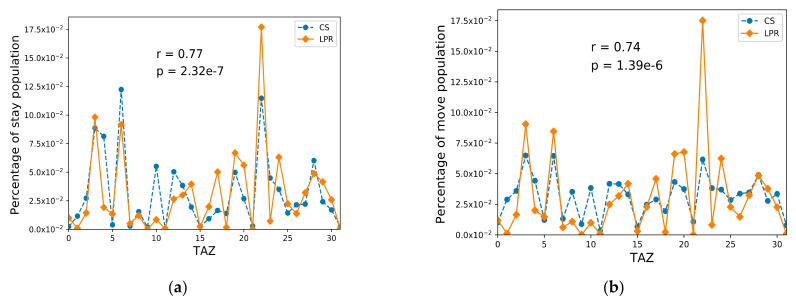
All-day population distributions with traffic analysis zone (TAZ) level. (**a**) All-day stay population distribution; (**b**) all-day move population distribution.

The OD recognition methods proposed in Section 3 were used to calculate the stay and move populations and to compare the urban population distribution of the two datasets. We used the 32 administrative streets/towns in Foshan as TAZs. The average area of the TAZ was 118.75 square kilometers, the average permanent population of the TAZ was 229,700, and the average registered population of the TAZ was 120,300. The results are shown in Figure 3. Figure 3a represents the all-day stay population distribution and Figure 3b denotes the all-day move population distribution.

As shown in Figure 3, although the two datasets were different, their user/vehicle distributions were basically the same. The calculated r value of the stay population was 0.77, and the *p*-value was 2.32 × 10^−7^. The r value of move population was 0.74, and the *p*-value was 1.39 × 10^−6^. Since the r values of the stay and move populations were both greater than 0.5 and less than 0.8, namely, 0.5 < |r| ≤ 0.8, the LPR and CS data were considered to be significantly correlated. Furthermore, the p-values of the stay and move populations were both less than 0.05. Therefore, the null hypothesis $H_{0}$ was rejected, and the LPR and CS data were considered to be significantly related, that is, the significance test was passed.

### 4.2. Correlation Analysis of Per-Hour Population Distribution

**Figure 4:**
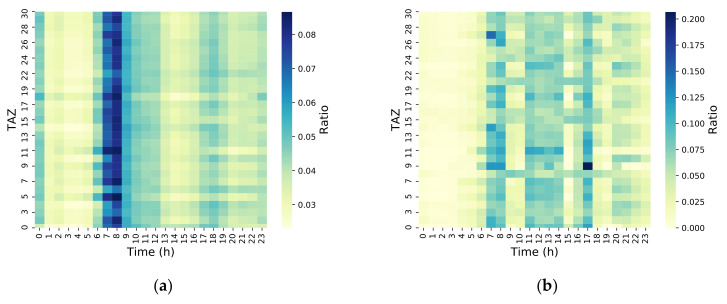
Hourly move population distribution for TAZ levels. (**a**) CS hourly move population distribution; (**b**) LPR hourly move population distribution.

**Figure 5:**
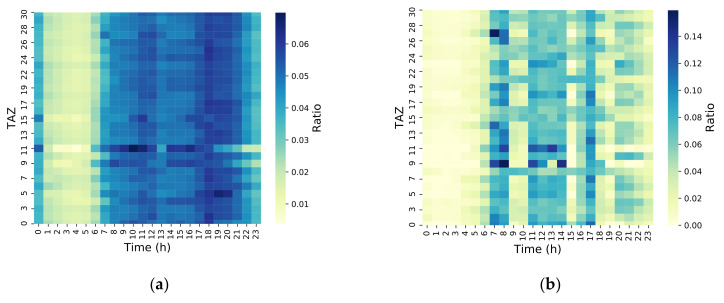
Hourly stay population distribution for TAZ levels. (**a**) CS hourly stay population distribution; (**b**) LPR hourly stay population distribution.

To further analyze the difference between the LPR and CS data in traffic patterns, the time scale was refined to the hour level. The results are shown in Figure 4 and Figure 5. The move and stay populations of the LPR and CS data were relatively small between 01:00 and 07:00. This showed that most mobile phones were turned off during this time period or remained at the same position for a long time. There were fewer vehicles traveling during this time period, which is consistent with typical human behavior. For the move population, the morning peak was identified readily by the CS data, while the evening peak was not as easily identified. However, the move population identified by the LPR produced a morning peak, noon peak, and evening peak, which showed that many workers went home for lunch at noon. This is also consistent with the typical travel behaviors in small cities. As this fraction of workers in the CS data was only a minority, there was no noon peak in the CS data. The stay population identified by the CS data was evenly distributed in other time periods except during the time window of 01:00–07:00, where the stay population was lower. The stay population identified by the LPR also produced a morning peak, noon peak, and evening peak. This was due to the mechanism of data generation. For CS, data were collected regardless of whether the user was traveling or not. For the LPR, data were collected only when the vehicle was traveling. Only when the vehicle was traveling could it be recognized as a stay. Therefore, this phenomenon agreed the typical behaviors of urban vehicle travel.

**Figure 6:**
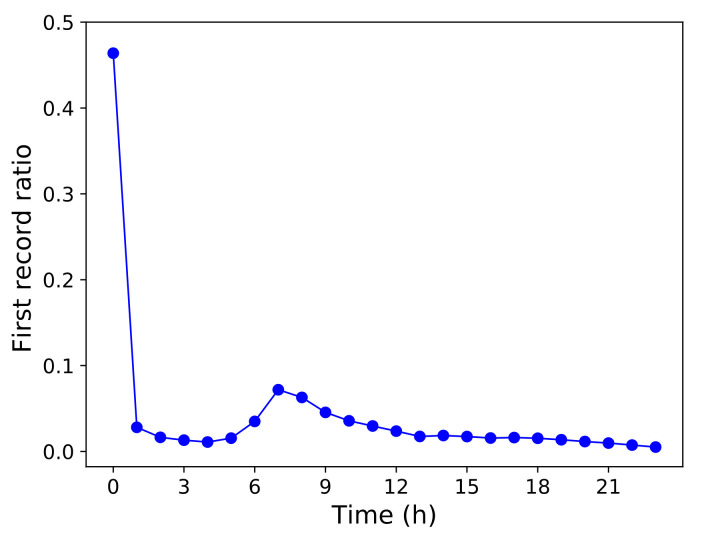
Proportional distribution of the first record at the hour level.

The stay and move populations identified by the CS were significantly higher in the 00:00–01:00 time window than in the 01:00–06:00 time window. In response to this problem, the time when the first CS record appeared was counted. The result is shown in Figure 6. The proportion of users whose records appeared between 00:00 and 01:00 was as high as 46.38%. The reason for this phenomenon may have been that (1) most mobile phone users do not sleep between 00:00 and 01:00, and (2) the data collection mechanism of the base station was involved.

**Figure 7:**
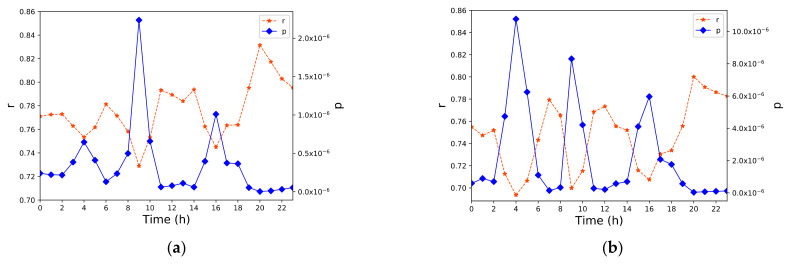
Population correlation analysis for TAZ levels. (**a**) Move population correlation; (**b**) stay population correlation.

The method proposed in Section 3.3 was used to calculate the correlation coefficients and *p*-values of the move and stay population distributions, and the results are shown in Figure 7. The correlation coefficient r of the move population was between 0.73 and 0.83, the r value of the No. 20 TAZ was as high as 0.83, which was in the highly correlated range. The remaining r values were in the range of 0.5 < |r| ≤ 0.8, which was a significant correlation. The *p*-values were all less than 0.05, so they passed the significance test. For the stay population, the r values were between 0.69 and 0.81, and the r value was greater than 0.8 in the No. 20 TAZ, which was in the highly correlated range. The remaining r values were between 0.5 and 0.8, corresponding to significant correlations. Similarly, all p-values were less than 0.05, so they also passed the significance test.

### 4.3. Travel Correlation Analysis

**Figure 8:**
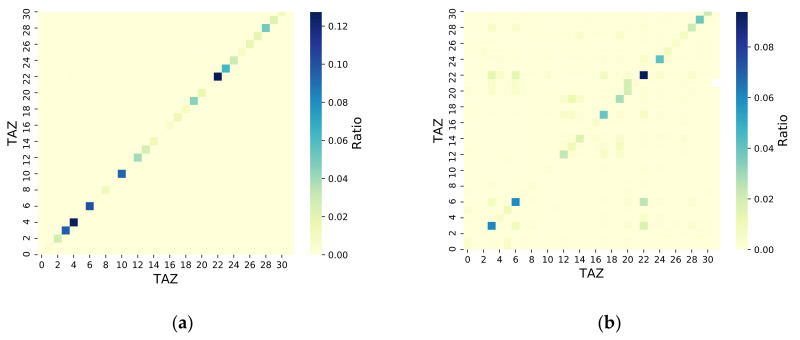
TAZ level origin–destination (OD) matrix from the LPR and CS data for one day. (**a**) CS OD matrix; (**b**) LPR OD matrix.

**Figure 9:**
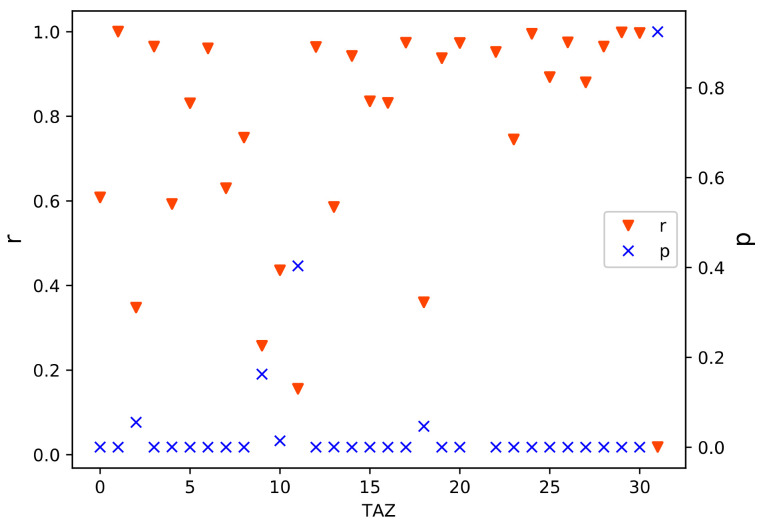
OD matrix correlation of LPR and CS data for TAZ levels.

Obtaining the OD matrix between TAZs is the basis for constructing a traffic model. Based on the OD recognition methods proposed in Section 3, the LPR and CS OD matrices were obtained for various TAZ levels. For a better comparison, similar to the population distribution, the ratio of the travel volume between TAZs to the total travel volume was used instead of an absolute number to analyze the differences in the OD values between the LPR and CS data. As shown in Figure 8, the OD matrices calculated by the LPR and CS data were very similar. Most users/vehicles moved in the same TAZ, and only a few moved across different TAZs. The OD matrix correlation coefficient r and *p*-value of the LPR and CS data were also calculated. The results, which are shown in Figure 9, showed that 59.36% of the r values were in the 0.8 < |r| ≤ 1 range, 18.75% were in the 0.5 < |r| ≤ 0.8 range, 9.38% were in the 0.3 < |r| ≤ 0.5 range, and 12.5% were in the 0.1 < |r| ≤ 0.3 range. All the *p*-values, except for those in the No. 2, 9, 11, and 31 TAZs, were less than 0.05. Therefore, in the No. 2, 9, 11, and 31 TAZs, the correlations between the CS and LPR data were poor. The reason for the poor correlations was that there were fewer LPR systems in these TAZs. Generally, LPR systems were installed densely in urban areas with heavy traffic and sparsely installed in urban suburbs. As a result, the number of vehicle trips in these TAZs was lower, and the individual characteristics of the high-frequency travelers readily affected the calculation results.

## 5. Conclusions

Compared with traditional survey methods, LPR and CS can quickly obtain the population distribution and travel patterns of urban residents. However, most of the analyses of the population distribution and urban traffic patterns are based on a single dataset. It is impossible to know whether there are differences in the analyses between different datasets. In addition, they usually do not distinguish between move population and stay population. It is critical to distinguish them in transportation research. As a result, there is no guarantee that the calculation results based on a single datum can meet the accuracy requirements of urban traffic analysis. To solve this problem, five working days of the LPR and CS data in Foshan City were examined, and different stay point recognition methods were used to calculate the population distribution and traffic patterns commonly used in urban traffic planning. For LPR data, different stay point recognition methods were designed according to whether the end point of the previous trip and the start point of the next trip are the same. For CS data, a spatiotemporal clustering algorithm based on time allocation is proposed to recognize stay points. Then, the correlations between the two datasets were analyzed. The results showed that there was high similarity between the population distribution and traffic patterns obtained from the two datasets. However, due to the different collection mechanisms of the two datasets, the identifications of the stay and move users/vehicles were slightly different. CS data should be collected regardless of whether the mobile phone user is traveling or not, while LPR data should only be collected when the vehicle is traveling. In addition, vehicle users who use mobile phones are only a minority of mobile phone users. Therefore, although there was a noon peak in the identification of vehicle users, since most mobile phone users do not travel at noon, there was no noon peak in the identification of the CS data. Moreover, the coverage of the LPR systems in some TAZs is low, and travel identification is greatly affected by individual travel, resulting in low correlations, such as No. 2, 9, 11, and 31 TAZs.

In most cases, the results of the population distribution and traffic pattern calculated between different traffic datasets and the extended conclusions were highly similar. The experiment results confirm that our proposed method can learn more temporal and spatial correlation among human mobility datasets to help urban transport planning. Our main contributions can be summarized as follows:

(1) A spatiotemporal clustering algorithm based on time allocation was proposed to identify stay points using cellular signaling data.
(2) Urban traffic patterns and population distribution were obtained from the perspective of multisource traffic data.
(3) The correlation between cellular signaling data and license plate recognition data was analyzed.
(4) The results revealed that cellular signaling data and license plate recognition data were significantly correlated in population distribution and urban traffic patterns.

Through this data mining and analysis, it is known that under the condition that the data quality is fully guaranteed, the fusion of different traffic datasets is more in line with urban traffic laws for analyzing population distribution and traffic patterns. It shows that the human travel data obtained through different data collection devices are not independent but are significantly correlated. In our future work, we will integrate more kinds of human mobility data for urban transport planning to support the development of intelligent transportation systems. Meanwhile, the integration of more datasets will lead to user privacy leakage, data privacy protection is one of our next research directions. Besides, the same TAZ may include office areas and residential areas. The population distribution of them must be different. Therefore, the population distribution characteristics of residential and office areas are also the future research direction.

## Figures and Tables

**Table 1:** Primary fields in the LPR dataset.

Fields	Example Value
Vehicle ID	9og0bdur59f8b096
Record time	2018-07-18 12:15:56
Address	Gaoming Road, Gaoming Bridge (East to West)
Drive direction	1
Detector ID	165897
Longitude	112.9625
Latitude	23.5614

**Table 2:** Instance of an individual’s cellular signaling (CS) data records in a day.

User ID	Cell ID	Record Time	Longitude	Latitude	Age	Gender	…
8s5h37jdrt4rf	E3g5y7k	00:14:21	112.1255	23.2351	25	M	…
8s5h37jdrt4rf	E3g5y7k	00:45:36	112.1255	23.2351	25	M	…
8s5h37jdrt4rf	E3g5y7k	00:58:13	112.5247	23.5832	25	M	…
8s5h37jdrt4rf	E3g5y7k	01:19:05	112.1255	23.2351	25	M	…
8s5h37jdrt4rf	…	…	…	…	…	…	…
8s5h37jdrt4rf	E3g5y7k	23:54:29	112.5321	23.6523	25	M	…

## Data Availability

The data presented in this study are available on request from the corresponding author. The data are not publicly available due to privacy.
